# SANAD: Single-label Arabic News Articles Dataset for automatic text categorization

**DOI:** 10.1016/j.dib.2019.104076

**Published:** 2019-06-04

**Authors:** Omar Einea, Ashraf Elnagar, Ridhwan Al Debsi

**Affiliations:** University of Sharjah, United Arab Emirates

**Keywords:** Arabic, Natural language processing, News articles, Single-label text classification

## Abstract

Text Classification is one of the most popular Natural Language Processing (NLP) tasks. Text classification (aka categorization) is an active research topic in recent years. However, much less attention was directed towards this task in Arabic, due to the lack of rich representative resources for training an Arabic text classifier. Therefore, we introduce a large Single-labeled Arabic News Articles Dataset (SANAD) of textual data collected from three news portals. The dataset is a large one consisting of almost 200k articles distributed into seven categories that we offer to the research community on Arabic computational linguistics. We anticipate that this rich dataset would make a great aid for a variety of NLP tasks on Modern Standard Arabic (MSA) textual data, especially for single label text classification purposes. We present the data in raw form. SANAD is composed of three main datasets scraped from three news portals, which are AlKhaleej, AlArabiya, and Akhbarona. SANAD is made public and freely available at https://data.mendeley.com/datasets/57zpx667y9.

**Table:** Specifications Table

Subject area	*Computer Science*
More specific subject area	*Arabic Language, Text Classification, Machine Learning, Natural Language Processing*
Type of data	*Text files*
How data was acquired	*By scraping news websites*
Data format	*Raw*
Experimental factors	*Texts are not cleaned or stemmed.* *Texts are organized into files; each file is one news article.* *Text files are grouped in folders where each folder corresponds to a category.*
Experimental features	*The dataset contains almost 200k articles, organized into a maximum of 7 categories.*
Data source location	*N/A*
Data accessibility	*Data is free, publicly available and can be downloaded from:*https://data.mendeley.com/datasets/57zpx667y9

**Table:** 

**Value of the data** • SANAD is the largest, to our knowledge, available and representative single-labeled Arabic dataset suitable for Text Classification as well as other NLP tasks. • SANAD offers up to seven main distinct categories, which are appropriately selected to eliminate any ambiguity and therefore making it robust for accurate text categorization. • SANAD caters for a wider variety of research needs consisting of 3 datasets compiled from different news sources and can be used as a benchmark. • In contrast with the few small available datasets, SANAD’s size makes it a suitable corpus for implementing both classical as well as deep learning models.

## 1 Data

**Fig. 1:**
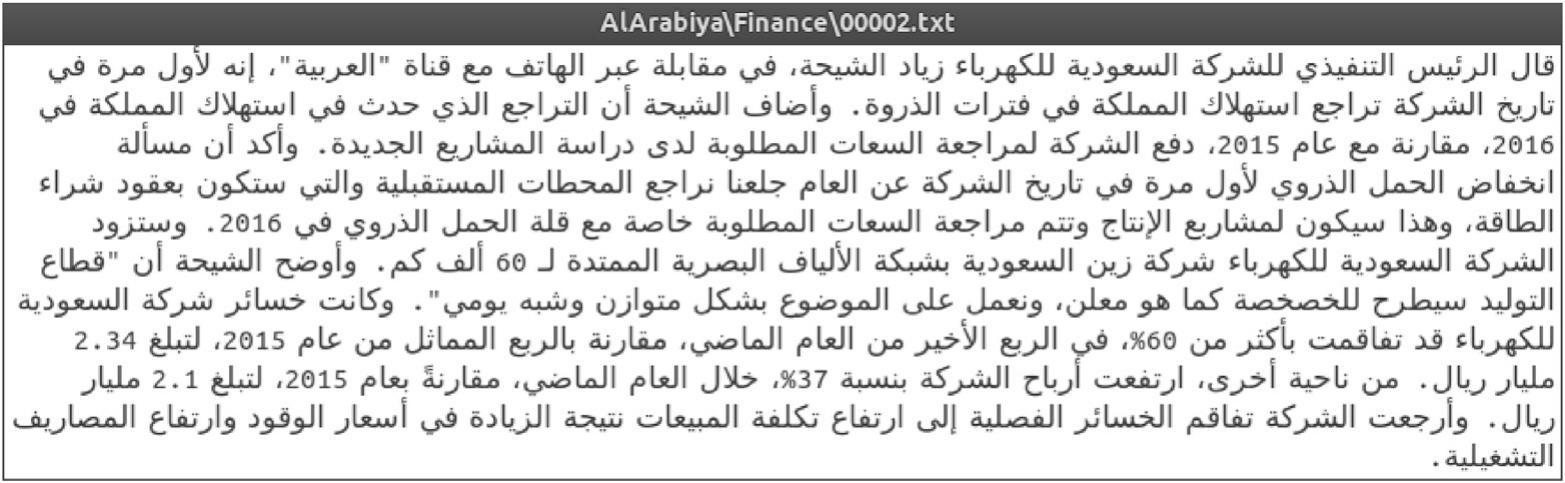
An example of an Article.

**Fig. 2:**
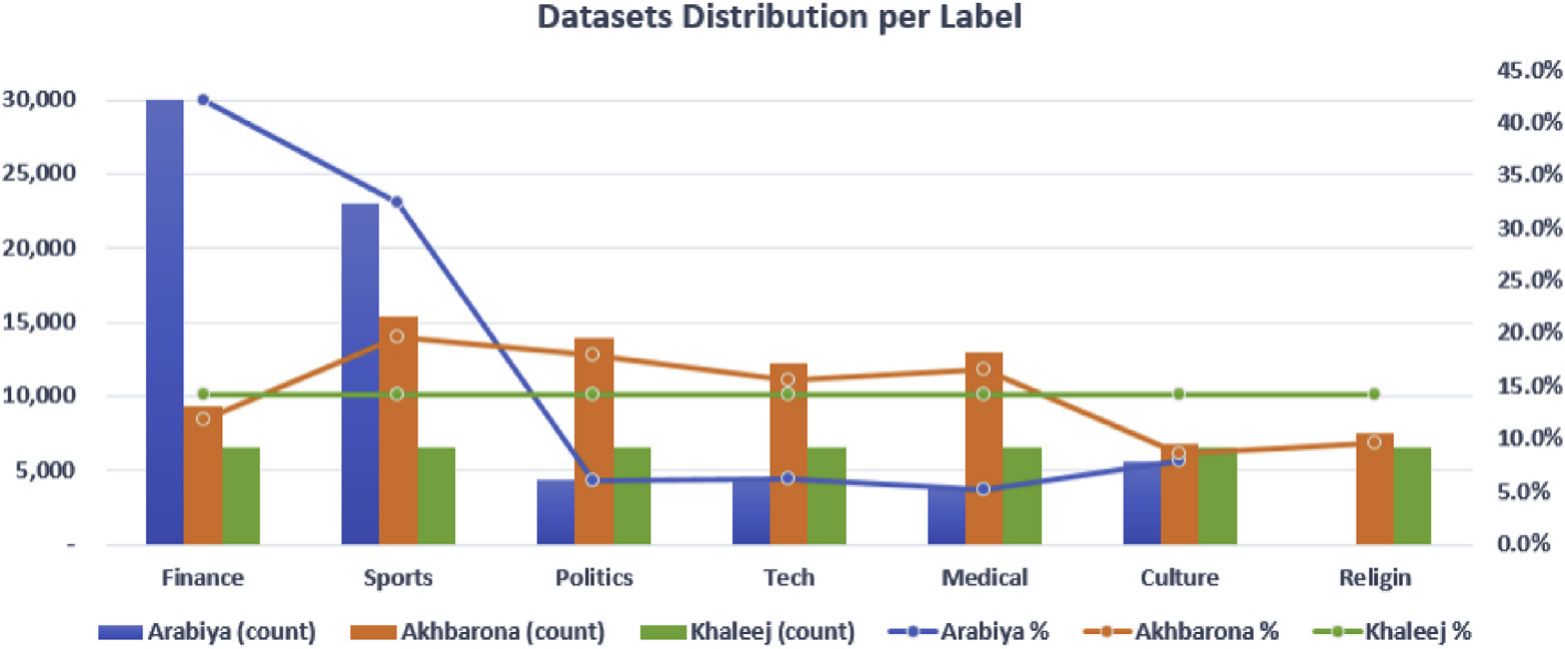
Distribution of articles per label for each dataset.

**Fig. 3:**
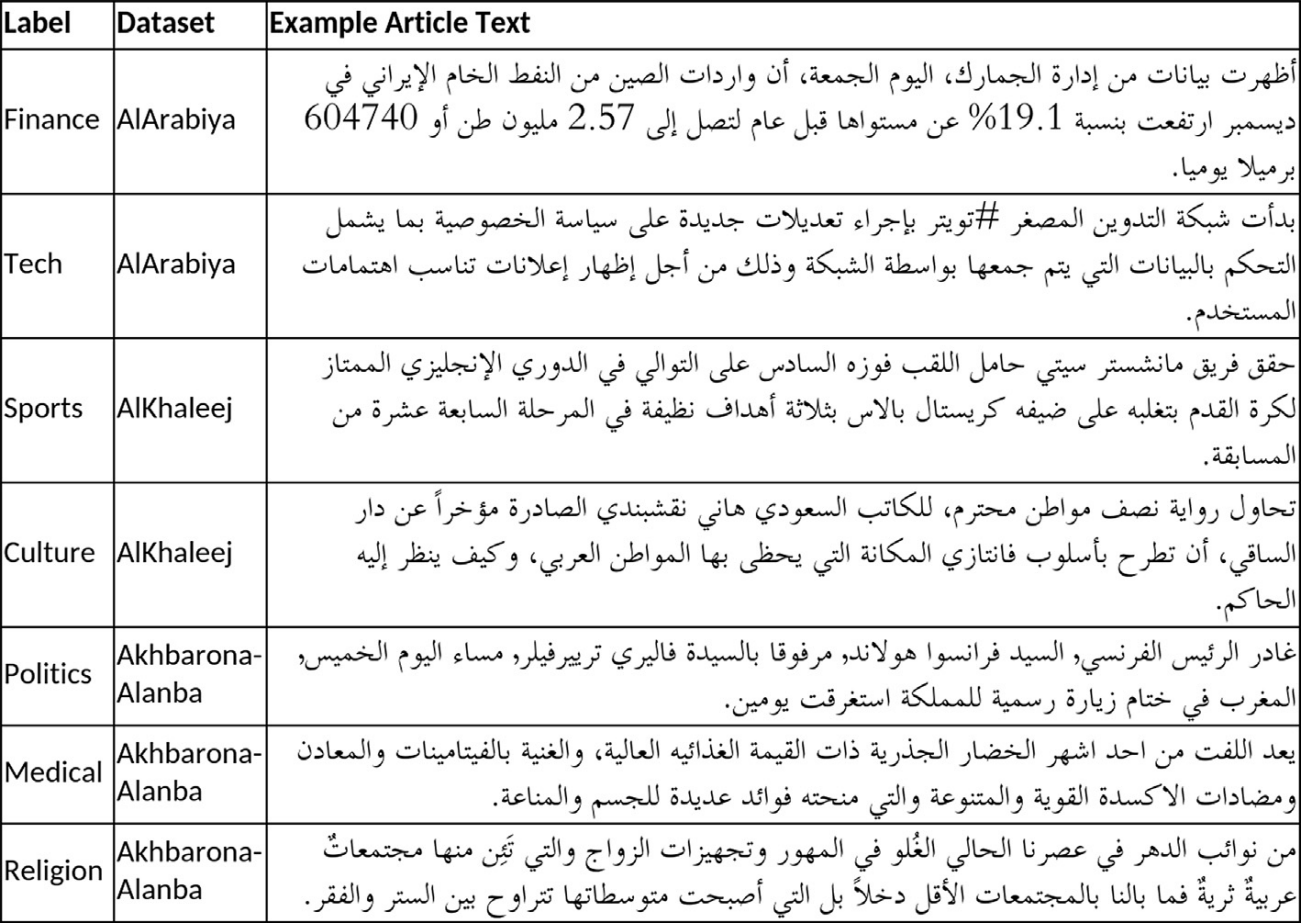
Illustrative examples from each category from the 3 datasets.

SANAD corpus is a large collection of Arabic news articles that can be used in several NLP tasks such as text classification and producing word embedding models. AlKhaleej and Akhbarona-Alanba datasets have seven categories, which are: Culture, Finance, Medical, Politics, Religion, Sports and Technology. As for AlArabiya dataset, it has six categories: Culture, Finance, Medical, Politics, Sports and Technology. SANAD has a total number of 194,797 articles categorized and formatted as shown in Fig. 1. In general, SANAD adopted the annotation of each article as appeared in its news portal source. Only one collection of articles is manually re-labeled to enrich the ‘politics’ category in AlArabiya dataset. The distribution of articles per category for each dataset is summarized in Table 1 and Fig. 2, Table 2 compares between SANAD and other already existing datasets, and a list of examples from the datasets is presented in Fig. 3.

**Table 1:** Distribution of articles per category.

Label	AlArabiya	Akhbarona-Alanba	AlKhaleej
Finance	30,076	9,280	6,500
Sports	23,058	15,377	6,500
Culture	5,619	6,746	6,500
Tech	4,411	12,199	6,500
Politics	4,368	13,979	6,500
Medical	3,715	12,947	6,500
Religion	–	7,522	6,500
Total	71,247	78,050	45,500

## 2 Experimental design, materials, and methods

The data is formatted as follows: there are three folders; a separate folder for each source of news website. Each folder has sub-folders that carry the title of the categories or labels. Each sub-folder contains a list of text files numbered sequentially, in which a file corresponds to one whole article. All articles are unique. Each article is kept in one sub-folder (i.e. under one label).

The data is kept in raw format as is; no cleaning, stemming or any type of pre-processing is applied after scraping. The articles contain some English symbols, punctuation, digits, and almost no Arabic diacritics. Fig. 1 shows an example of an article that is categorized as “Finance” and belongs to “AlArabiya” dataset.

The distributions of all articles per category (aka label) per dataset in terms of count and percentages are depicted in Fig. 2. While AlArabyia and Akhbarona-Alanba datasets are unbalanced, AlKhaleej dataset is kept balanced. The detailed number of articles per category for each dataset is shown in Table 2.

**Table 2:** Comparison between SANAD and other datasets.

Dataset	# of Categories	Categories	# of Articles	Avg. Articles per Category
Khaleej-2004	4	International News, Local News, Economy, Sports	5,690	1,423
Watan-2004	6	International News, Local News, Economy, Sports, Culture, Religion	20,291	3,382
SL-RTANews	40	Football, Syrian Crisis, Armed Groups, Oil Markets, Other Sports, Syrian Rebels, Refugees, etc.	23,837	596
SANAD	7	Finance, Sports, Culture, Technology, Politics, Medical, Religion	194,797	27,828

We came up with the SANAD abbreviation as it has the meaning of support in Arabic language. The articles were collected using Python scripts written specifically for scraping three popular news portals. Those scripts load the list of portal’s articles, enter each article’s page and get its text and tags. The data collection procedures are described below for each of the news portals:

1. AlArabiya

The main website [5], has two subdomains: ‘alhadath’ and ‘aswaq’. After scraping the articles, we filtered them to make six categories as mentioned above. We adopted the same categories or tags of the news portal. The ‘Religion’ category is not listed among the categories of the news source and therefore it is dropped. After examining the content of categories, one category tagged with ‘Iran’ is manually merged with the ‘Politics’ category. This is because it is most relevant to this class and to provide a better balance among the dataset's categories. We collected a set of 72k articles since October 2012 until April 2018 (scarping time). We applied some further data filtering by removing irrelevant articles that cannot fit in any of the six categories; such articles were originally tagged from the source with ‘Miscellaneous’ label. The resulting distribution of the six categories (Table 1), which ranges between 5% and 45%, is shown in Fig. 2.

2. AlKhaleej

We scraped all articles from this news portal [6], since 2008 and until 2018. We collected more the 4GB of textual data. However, most articles on this website were not categorized or had a vague label. As a result, we only limited the data to the aforementioned seven categories and populated each category with a reasonable number of articles to serve text classification tasks. We made this dataset a balanced one by limiting the number of articles to 6500 articles (Table 1) in line with the minimum populated category. Fig. 2 reflects this distribution.

3. Akhbarona-Alanba

Similar to AlArabyia, we collected all relevant articles from Akhbarona news portal [7], for the seven mentioned categories. One category, ‘Religion’, had half as much as other categories did. Thus, to enrich this category, we collected the remaining half of that category (that is 5% of the dataset) from a similar newspaper website, which is Alanba news portal [8]. The distribution of articles (Table 1) ranges between 9% and 20% for each. See Fig. 2 for details. We collected a set of 78k articles since January 2011 until October 2018 (scraping time).

SANAD comprises of the above three datasets, which makes it the largest, to our knowledge, available and representative corpus. In contrast with other few available datasets such as those used in [2], [3], [4], SANAD is large enough to enable researchers to implement classical and deep learning models for text classification as it is the case in [1], which used for sentiment classification. Few similar datasets already exist but are not comparable in size and have less tags. For example, ‘Khaleej-2004’ [9], consists of around 6k articles distributed into four categories. The ‘Watan-2004’ [10], comprises of around 20k and six labels. The recently reported SL-RTAnews for single-label classification [11], contains 23,837 articles distributed over 40 categories. However, the distribution of articles in its categories is biased for few main categories if we apply some filtering on the size of categories. For example, if a minimum of 1000 articles is required for training and testing of a Deep Neural Network (DNN) based classifier then only 4 categories can be used.

SANAD offers around 200k articles spanning seven categories. Besides, the articles are tagged with the most relevant category, while exiting datasets have ambiguous categories such as ‘International News’ or ‘Local News’. In contrast with the rest, SANAD is a suitable dataset for implementing Deep Learning classifiers. Table 2 details the statistics on each of the available free datasets. Fig. 3 contains illustrative examples of each category from the 3 datasets of SANAD.
